# Prognostic significance of positive family history in outcomes after coronary artery bypass grafting: Do we need to update our assumptions?

**DOI:** 10.1186/s13019-022-01836-4

**Published:** 2022-04-27

**Authors:** Hamed Tavolinejad, Sina Rashedi, Seyyed Mojtaba Ghorashi, Masih Tajdini, Saeed Sadeghian, Mina Pashang, Arash Jalali, Abbas Salehi Omran, Jamshid Bagheri, Abbasali Karimi, Mahmoud Shirzad, Mehdi Mehrani, Kaveh Hosseini

**Affiliations:** 1 Tehran Heart Center, Cardiovascular Diseases Research Institute, Tehran University of Medical Sciences, Tehran, Iran; 2 Non-Communicable Diseases Research Center, Endocrinology and Metabolism Population Sciences Institute, Tehran University of Medical Sciences, Tehran, Iran; 3 Cardiac Primary Prevention Research Center, Cardiovascular Diseases Research Institute, Tehran University of Medical Sciences, Tehran, Iran

**Keywords:** Family history of cardiovascular disease, Coronary artery bypass, Mortality, Coronary artery disease, Cardiovascular disease

## Abstract

**Background:**

Recent research suggests a protective role for positive family history of premature cardiovascular disease (FHpCVD) in patients undergoing coronary artery bypass grafting. We aimed to further investigate this unlikely association.

**Methods:**

In this registry-based cohort study, patients who underwent first-time non-emergent coronary bypass surgery at Tehran Heart Center between 2007 and 2016 were included. Patients with and without FHpCVD were compared in terms of all-cause mortality and first non-fatal cardiovascular events (CVEs) comprising non-fatal acute coronary syndrome, non-fatal stroke or transient ischemic attack, and repeat coronary revascularization.

**Results:**

A total of 13,156 patients were included (mean age 60.83 ± 9.57, 74.5% male), among which 2684 (20.4%) patients had FHpCVD. Median follow-up was 77.7 months. FHpCVD was weakly associated with reduced all-cause mortality using inverse probability weight (IPW) method (hazard ratio [HR] = 0.853; 95% confidence interval [CI] 0.730–0.997; P = 0.046), and not associated with non-fatal CVEs considering death as the competing event (sub-distribution HR [SHR] = 1.124; 95% CI 0.999–1.265; P = 0.053). Within a subgroup of patients without previous myocardial infarction or revascularization (7403 cases; 56.3%), FHpCVD was associated with lower mortality (HR = 0.700; 95% CI 0.548–0.894; P = 0.004) and higher non-fatal CVEs (SHR = 1.197; 95% CI 1.019–1.405; P = 0.028), whereas among patients with previous coronary events, there was no association between FHpCVD and outcomes.

**Conclusions:**

FHpCVD was associated with lower all-cause mortality but higher non-fatal CVEs, especially in those without prior coronary events. Such discordance calls for caution in assuming a protective role for FHpCVD. The prognostic significance of FHpCVD needs further evaluation among surgical patients.

## Background

Family history of premature cardiovascular disease (FHpCVD) in first-degree relatives is an established risk factor for the first occurrence of adverse cardiovascular events (CVEs) [1, 2]. The presence of FHpCVD is associated with incident myocardial infarction (MI), stroke, and an almost 50% higher risk of CVD-related mortality in lifetime [2, 3]. In individuals without CVD, FHpCVD is considered a risk enhancer that can guide decisions in primary prevention, such as for antihypertensive and lipid-lowering interventions [4]. On the other hand, among patients with established CVD, the utility of FHpCVD as a prognostic marker is less recognized, and the available studies have shown mixed results.

A recent study has shown a paradoxically protective role for FHpCVD in patients undergoing coronary artery bypass grafting (CABG) surgery [5]. Prediction of long-term outcomes after CABG surgery is particularly important since such individuals have a high-risk profile which should be considered in clinical decision making [6]. Furthermore, studies among patients with CVD have reported better survival rates associated with FHpCVD [7, 8]. In this context, further investigation is warranted to clarify the potential protective or risk enhancing role of FHpCVD among patients undergoing CABG. In the current study, we aimed to evaluate the association of FHpCVD and cardiovascular outcomes in a large population of patients who underwent isolated CABG at a tertiary center.

## Materials and methods

### Design and population

In this registry-based cohort study, all patients who underwent CABG at Tehran Heart Center [9, 10] between 2007 and 2016 were screened. Patients who had isolated first-time non-emergent CABG with available data regarding FHpCVD were included. Comparisons were made between patients with and without the presence of FHpCVD. FHpCVD was determined by patient self-report and medical records at the time of surgery and was defined as the occurrence of coronary events including cardiac death, MI, or coronary revascularization utilizing CABG or percutaneous coronary intervention (PCI) in a male first-degree relative before the age of 55 years and/or a female first-degree relative younger than 65 years [11, 12]. Furthermore, subgroup analyses were performed to assess the association of FHpCVD and outcomes among patients with and without a past history of MI or coronary revascularization. Prior CVEs (MI or coronary revascularization) were defined based on medical records of the participants.

The study protocol was approved by the Research Ethics Committee of School of Medicine, Tehran University of Medical Sciences (Ethics code:IR.TUMS.THC.REC.1399.045), and was conducted according to the principles of the declaration of Helsinki and its later amendments. Written informed consent was obtained from all participants at the time of their inclusion in the CABG registry.

### Follow-up and outcomes

Patients were invited to participate in clinic visits at 1-, -6, and -12 months following surgery and each year thereafter according to our center's follow-up protocol. Experienced practitioners visited patients, and at each visit, data regarding symptoms, cardiovascular risk factors (hypertension, dyslipidemia, diabetes mellitus, obesity, tobacco smoking, and opium consumption) and their control status, FHpCVD, anthropometric, laboratory, and echocardiographic findings were evaluated. Obesity was defined by a body mass index ≥ 30 kg/m^2^. In case of inability to participate in clinic visits, the follow-up was accomplished through a nurse-led telephone call. The vital status of participants and the occurrence of CVEs were assessed at each follow-up interval. Non-fatal CVEs were assessed based on patient medical records obtained through visits or phone calls.

The outcomes of interest were death due to all-causes and a composite secondary outcome of non-fatal CVEs, comprised of non-fatal acute coronary syndromes (ACS), non-fatal stroke or transient ischemic attack (TIA), and repeat coronary revascularization via PCI or redo-CABG. Development of ACS was identified according to the final diagnosis in the documented medical records, including ischemia-related electrocardiographic abnormalities and laboratory tests. Stroke and TIA were adjudicated according to the documented clinical manifestations of sudden onset neurological impairment diagnosed by the treating physician, along with neuro-imaging records ruling out possible differential diagnoses.

### Statistical analysis

Normality of continuous variables was assessed using histograms, central tendency and dispersion measures. Normally distributed variables were described as mean ± standard deviation, while skewed data were presented as median (25th and 75th percentiles). Continuous variables were compared using independent samples T or Mann–Whitney U-test. Categorical variables were expressed as frequency (percentage), and were compared by the chi-squared test. The length of follow-up was assessed by the reverse Kaplan–Meier method to calculate median follow-up with the corresponding 95% confidence interval (CI) [13].

The positive- and negative-FHpCVD groups were balanced using stabilized inverse probability weights (IPWs) calculated from propensity scores to account for effects of potential confounders (including age, gender, body mass index, dyslipidemia, hypertension, diabetes mellitus, chronic obstructive pulmonary disease, renal failure, tobacco smoking, opium consumption, previous PCI, previous MI, ejection fraction, left main coronary disease, off-pump CABG, and the number of graft). Opium consumption was adjusted for because of recent data demonstrating worse outcomes after CABG in opium consumers [14]. A weighted (based on IPWs) Cox proportional hazards model was applied to evaluate the independent effect of positive FHpCVD on all-cause mortality and non-fatal CVEs, and the effects were reported as hazard ratio (HR) with 95% CI. Furthermore, non-fatal CVEs were considered in a competing risk setting regarding death before non-fatal CVEs as a competing event. The adjusted effects were calculated through a competing risks regression model and were reported as sub-distribution HR (SHR) and corresponding 95% CI.

To evaluate unmeasured confounding, E-values were calculated for the investigated associations by using and online tool [15, 16]. By definition, E-value is the minimum strength of an association between a confounder with both the exposure and the outcome, in terms of relative risk and beyond the already measured covariates, that can potentially explain away the observed association [15].

In order to further explore the impact of FHpCVD, the analyses were repeated in two subgroups of patients with and without at least one prior event of MI or PCI. Analyses were conducted using Stata statistical software release 14.0 (College Station, TX: StataCorp LP).

## Results

### Study population

Among 13,385 eligible patients, 229 (1.7%) were lost to follow up. Fifteen patients had moved abroad, 24 were unwilling to participate in follow-up sessions, and 190 did not answer the repeated phone calls. Ultimately, 13,156 patients who underwent isolated first-time non-emergent CABG at our center were included (mean age 60.83 ± 9.57, 74.5% male), among whom 2684 (20.4%) patients had FHpCVD. Patients with FHpCVD were younger (57.25 vs. 61.75 years; P < 0.001), more likely female (30.0% vs. 24.4%; P < 0.001), had higher rates of obesity (28.3% vs. 23.3%; P < 0.001), dyslipidemia (62.0% vs. 56.8%; P < 0.001), prior PCI (8.8% vs. 7.1%; P = 0.003), and higher ejection fraction (47.60% vs. 46.67%; P < 0.001). Table 1 summarizes the baseline characteristics of the study population.

**Table 1:** Baseline characteristics

Variable	Total (n = 13,156)	With FHpCVD (n = 2684)	Without FHpCVD (n = 10,472)	P-value
Age (years)	60.83 ± 9.57	57.25 ± 9.33	61.75 ± 9.42	**< 0.001**
Male gender	9798 (74.5%)	1878 (70.0%)	7920 (75.6%)	**< 0.001**
BMI (kg/m^2^)				
< 18.5	89 (0.7%)	17 (0.6%)	72 (0.7%)	0.765
18.5–24.9	3790 (28.9%)	684 (25.6%)	3106 (29.8%)	**< 0.001**
25–29.9	6044 (46.1%)	1213 (45.4%)	4831 (46.3%)	0.426
≥ 30	3183 (24.3%)	756 (28.3%)	2427 (23.3%)	**< 0.001**
Dyslipidemia	7609 (57.9%)	1664 (62.0%)	5945 (56.8%)	**< 0.001**
Hypertension	7050 (53.6%)	1460 (54.4%)	5590 (53.4%)	0.347
Diabetes mellitus	5034 (38.3%)	987 (36.8%)	4047 (38.7%)	0.073
COPD	317 (2.4%)	52 (2.0%)	265 (2.6%)	0.079
Renal failure	268 (2.0%)	45 (1.7%)	223 (2.1%)	0.134
Tobacco smoking				0.296
Previous smoking	2464 (18.8%)	505 (18.9%)	1959 (18.8%)	
Current smoking	2522 (19.2%)	542 (20.2%)	1980 (19.0%)	
Opium consumption	1848 (14.3%)	382 (14.5%)	1466 (14.3%)	0.824
Previous PCI	977 (7.4%)	235 (8.8%)	742 (7.1%)	**0.003**
Previous MI	5240 (39.8%)	1090 (40.6%)	4150 (39.6%)	0.354
LVEF (%)	46.86 ± 9.61	47.60 ± 9.56	46.67 ± 9.61	**< 0.001**
Left main coronary disease	1022 (7.8%)	188 (7.0%)	834 (8.0%)	0.098
Off-pump surgery	717 (5.4%)	130 (4.8%)	587 (5.6%)	0.121
Graft number	3 (3–4)	3 (3–4)	3 (3–4)	0.395

Data are represented as mean ± standard deviation, median (interquartile range), or numbers (%); P-values < 0.05 are shown in bold font

BMI, body mass index; COPD, chronic obstructive pulmonary disease; FHpCVD, family history of premature cardiovascular disease; LVEF, left ventricular ejection fraction; MI, myocardial infarction; PCI, percutaneous coronary intervention

### Endpoints

**Fig. 1:**
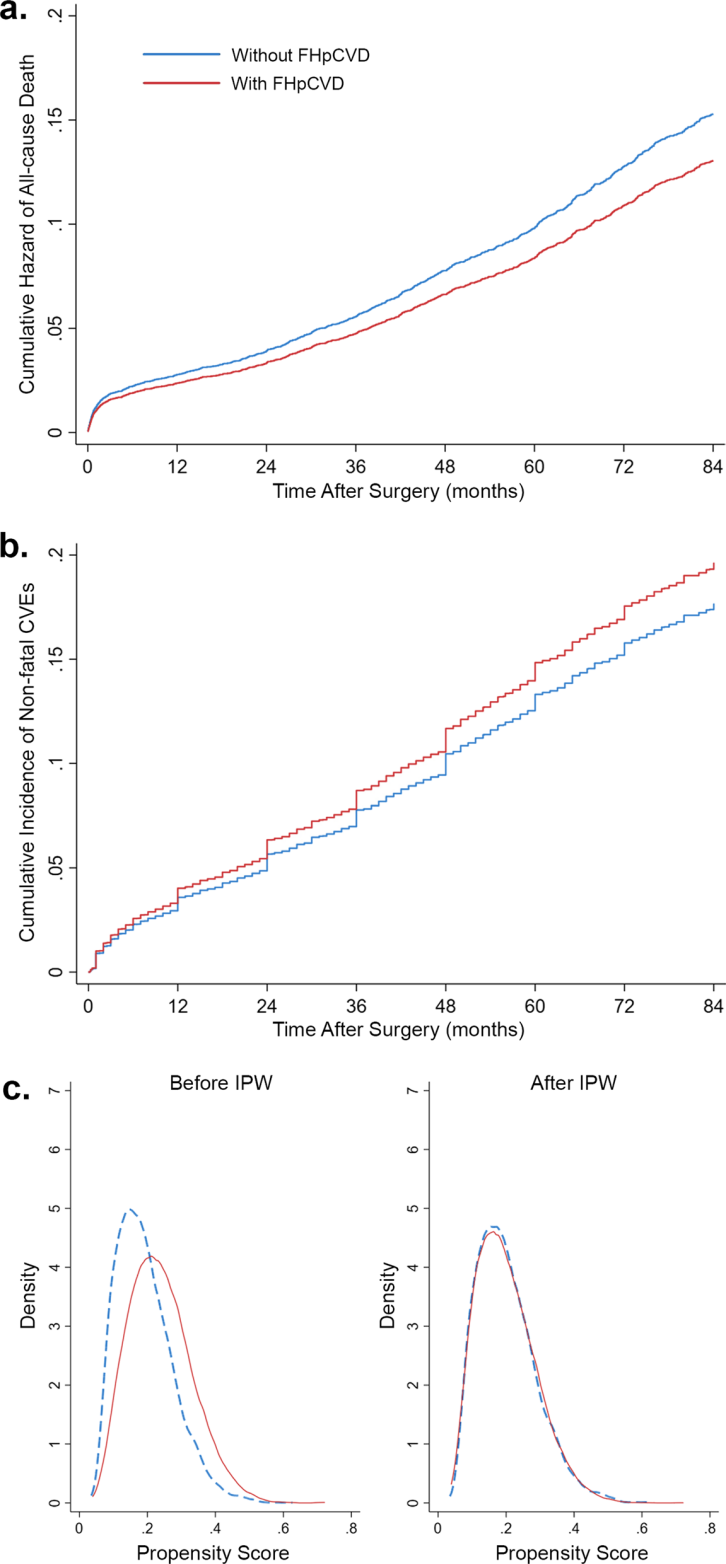
Association of family history of premature cardiovascular disease (FHpCVD) with outcomes after inverse probability weighting (IPW); **a** all-cause mortality, **b** non-fatal cardiovascular events (CVEs) with the competing event of death, **c** propensity score adjustments

The median follow-up duration was 77.7 months (95% CI 76.6–78.7 months). Overall, 1878 patients (14.3%) died during the follow-up period (Table 2). Unadjusted analysis revealed that FHpCVD was associated with a reduced risk of all-cause mortality (HR = 0.635, [95% CI 0.560–0.719]; P < 0.001). This effect was reduced but remained statistically significant after IPW-adjusted analysis was performed to account for presumed confounders (HR = 0.853, [95% CI 0.730–0.997]; P = 0.046; Fig. 1a, c). The E-values for the point estimate and upper bound CI for all-cause mortality were 1.62 and 1.06, respectively.

**Table 2:** Frequency of outcomes

Total (n = 13,156)	With FHpCVD (n = 2684)	Without FHpCVD (n = 10,472)
All-cause mortality	295 (11%)	1583 (15.1%)
Non-fatal CVEs	517 (19.3%)	1583 (15.1%)
ACS	371 (13.8%)	1087 (10.4%)
Stroke/TIA	72 (2.7%)	279 (2.6%)
Repeat revascularization	74 (2.8%)	217 (2.1%)

With prior MI or PCI (n = 5753)	With FHpCVD (n = 1214)	Without FHpCVD (n = 4539)
All-cause mortality	167 (13.7%)	709 (15.6%)
Non-fatal CVEs	230 (18.9%)	703 (15.5%)
ACS	154 (12.7%)	472 (10.4%)
Stroke/TIA	41 (3.3%)	129 (2.8%)
Repeat revascularization	35 (2.9%)	102 (2.3%)

No prior MI or PCI (n = 7403)	With FHpCVD (n = 1470)	Without FHpCVD (n = 5933)
All-cause mortality	128 (8.7%)	874 (14.7%)
Non-fatal CVEs	287 (19.5%)	880 (14.8%)
ACS	217 (14.8%)	615 (10.4%)
Stroke/TIA	31 (2.1%)	150 (2.5%)
Repeat revascularization	39 (2.6%)	115 (1.9%)

Data are represented as number (percentage)

FHpCVD, family history of premature cardiovascular disease; CVE, cardiovascular event; ACS, acute coronary syndrome; TIA, transient ischemic attack; MI, myocardial infarction; PCI, percutaneous coronary intervention

A total of 2100 (16.0%) non-fatal CVEs occurred during the follow-up (Table 2). In contrast to the outcome of all-cause mortality, patients with FHpCVD were more likely to experience non-fatal CVEs in unadjusted analysis (HR = 1.185, [95% CI 1.073–1.308]; P = 0.001); however, this difference did not show statistical significance after IPWs and the competing risk of death were considered (SHR = 1.124, [95% CI 0.999–1.265]; P = 0.053; Fig. 1b, c). Because this association did not meet statistical significance, E-values were not calculated.

### Subgroups

**Fig. 2:**
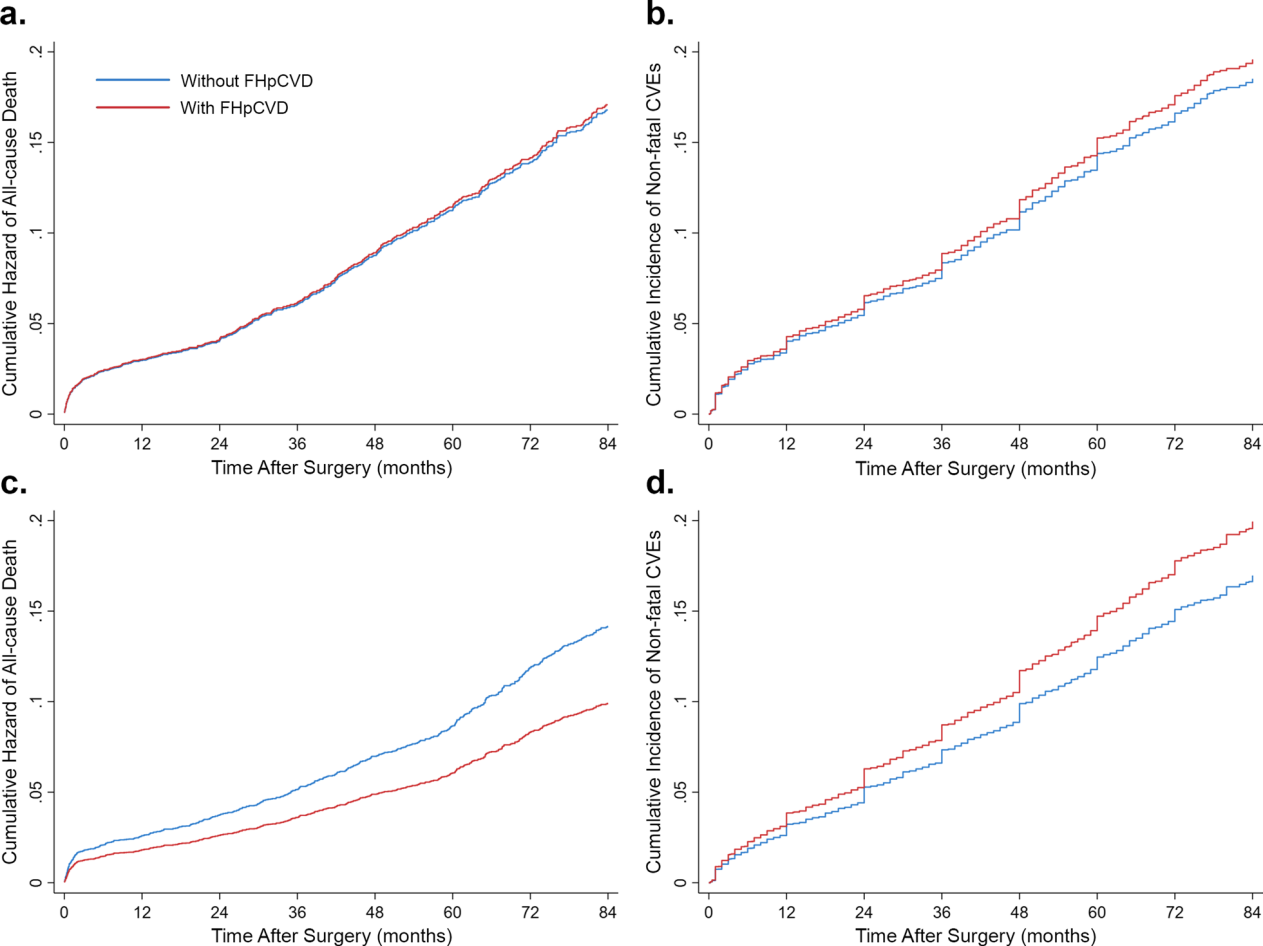
Association of family history of premature cardiovascular disease (FHpCVD) with outcomes after inverse probability weighting (IPW) in the subgroup with previous myocardial infarction (MI) or revascularization **a** all-cause mortality, **b** non-fatal cardiovascular events (CVEs) with the competing event of death; and among patients without previous MI or revascularization in terms of **c** all-cause mortality, **d** non-fatal cardiovascular events (CVEs) with the competing event of death

The subgroup of patients with at least one prior event of MI or PCI accounted for 5753 (43.7%) participants, among which 1214 (21.1%) patients had FHpCVD. In this subset of cases, patients with FHpCVD had a lower risk of mortality (HR = 0.789, [95% CI 0.666–0.934]; P = 0.006) and a higher risk of non-fatal CVEs (HR = 1.149, [95% CI 0.990–1.333]; P = 0.068) in unadjusted analysis; however, no statistically significant difference was detected after IPW adjustment for all-cause mortality (HR = 1.017, [95% CI 0.826–1.253]; P = 0.871; Fig. 2a) or non-fatal CVEs (SHR = 1.064, [95% CI 0.892–1.271]; P = 0.489; Fig. 2b).

The number of patients without a prior history of MI or PCI was 7403 (56.3%), among whom 1470 (19.8%) had FHpCVD. In this subgroup, patients with FHpCVD had significantly lower mortality rates both in unadjusted (HR = 0.498, [95% CI 0.413–0.600]; P < 0.001) and IPW-adjusted analysis (HR = 0.700, [95% CI 0.548–0.894]; P = 0.004; Fig. 2c). Nevertheless, patients with FHpCVD were at increased risk of non-fatal CVEs both in unadjusted analysis (HR = 1.212, [95% CI 1.060–1.385]; P = 0.005) and after accounting for IPWs and the competing risk of death (SHR = 1.197, [95% CI 1.019–1.405]; P = 0.028; Fig. 2d).

## Discussion

In this large cohort of patients undergoing CABG, FHpCVD was unexpectedly associated with lower all-cause mortality after a median of 6.5 years of follow-up; however, this association was weak when potential confounders were considered. On the other hand, patients with FHpCVD experienced a higher occurrence of non-fatal CVEs,which, after applying IPW and considering the competing risk of death, marginally missed the pre-defined level of statistical significance. We found no associations between FHpCVD and outcomes within patients who had had an event prior to the index operation. Importantly, however, among patients who did not have a history of MI or PCI at the time of CABG, FHpCVD was associated with a 30% relatively lower incidence of all-cause mortality and a 20% relatively higher rate of non-fatal CVEs after IPW and competing risk analysis.

The observation that the rate of non-fatal CVEs is not lower among patients with FHpCVD, and observing a trend that may suggest an even higher incidence of non-fatal CVEs among this group, calls for caution in interpreting the apparent protective role of FHpCVD in terms of all-cause mortality. FHpCVD is a risk factor for CVEs that may, in turn, result in mortality [1, 2]; therefore, supposing FHpCVD is not associated with a lower rate of CVEs in this patient population, questions the assumption of FHpCVD being protective against mortality.

### Prior evidence among patients with established CVD

Among patients undergoing CABG, a recent study by Ruttmann et al. [5]. found FHpCVD to be associated with improved eight-year survival by an adjusted HR of 0.67 (95% CI: 0.50–0.90). A composite of MI, stroke, cardiovascular death, or repeat revascularization was reduced in the presence of FHpCVD (HR = 0.73; 95% CI: 0.68–0.89). This study had a sample of 2535 patients with first-time, non-emergent CABG who were younger than 60 years of age. Apart from only focusing on younger CABG patients (termed premature CVD patients), the authors also excluded patients with single-vessel disease. The definition of self-reported parental FHpCVD was based on the Framingham offspring study criteria. In contrast to our findings, this study identified FHpCVD in 54% of participants, which is probably due to different eligibility criteria. Our FHpCVD rate of 20.4% is closer to previous studies in patients with CVD [7, 8, 17–20] and in healthy subjects [1, 21]. On the other hand, the prevalence of FHpCVD as a risk factor in patients with premature CVD has been estimated to be around 40% [22], which is obviously higher than the total (premature and non-premature combined) population. In the study by Ruttman et al. 40% of patients with FHpCVD and 23% of those without FHpCVD had a history of previous MI. Other baseline characteristics were comparable with our study. Patients with FHpCVD were younger and more likely to have hypertension, familial dyslipidemia, and a history of MI or cardiac interventions, while the rate of smoking, diabetes mellitus, peripheral artery, kidney, lung, and cerebrovascular diseases was lower in the presence of FHpCVD.

While data about patients undergoing elective CABG is scarce, several studies have investigated the prognostic role of FHpCVD among patients with established CVD. Abdi-Ali et al. reported that in patients with angiographically proven coronary disease, FHpCVD was associated with a 23% relative risk reduction of all-cause mortality over 5.6 years [7]. In a comparable subgroup analysis to our study, they found that among both subgroups with or without previous MI or revascularization, FHpCVD was associated with lower all-cause mortality; neverthelss, this association was weaker within the subgroup who had prior MI or revascularization. Studies among patients with ACS and MI, although heterogenous in their population, definition of FHpCVD, and outcomes, have also reported the same paradoxical association between FHpCVD and better prognosis [8, 17, 18, 20].

Against such background of previous research, the most notable findings in our study are the weakened association between FHpCVD and lower mortality after adjustment, similar, or perhaps even higher rate of non-fatal CVEs with a competing risk of death among patients with FHpCVD, and the clear distinction between patients with and without previous MI or revascularization in terms of the prognostic importance of FHpCVD. These findings make the existence of a causal link between FHpCVD and lower mortality less likely.

### Interpretation of findings

In explaining the association of FHpCVD and all-cause mortality, the role of confounders should be considered. Patients with FHpCVD may require CABG at a younger age and have fewer comorbidities and risk factors at the time of surgery—a point which is suggested by the weakened association in the IPW-adjusted analysis. When assessing the significance of unmeasured confounding for the association of FHpCVD and mortality by using E-values, we observed that a confounder (such as lifestyle or non-cardiovascular morbidity) with a relative risk of 1.06 (associated with both all-cause mortality and FHpCVD) can potentially alter the results of this analysis for the CI to include unity.

Among patients without established CVD, the higher risk associated with FHpCVD is independent of conventional CVD risk factors and is not mediated by metabolic comorbidities [3, 23]. This means that having fewer risk factors alone may not fully explain a better survival rate for individuals with FHpCVD. Patients with FHpCVD are shown to have a higher level of physical activity, better diet [3], better adherence to lifestyle modification [24], and earlier presentations to seek medical care [19, 25], all of which can result in a better survival rate. Patients with FHpCVD could receive preferential treatment, as Abdi-Ali et al. found a modestly higher rate of medication use among this group [7]. Other potential factors that have not been considered in the available studies with retrospective longitudinal cohort designs are long-term adherence to medical interventions, self-education, and the presence of non-cardiovascular comorbidities.

Another probable explanation for the association between FHpCVD and mortality in the literature is the existence of a collider in studies that focus on patients with CVD [26]. Two causal pathways can be considered:

1. FHpCVD: indication of CABG: mortality/survival.
2. Other risk factors (comorbidities, lifestyle, treatments, etc.): indication of CABG: mortality/survival.

Between the above pathways, the indication of CABG can be a collider. Moreover, survivorship bias can affect the results, as high-risk patients with FHpCVD may have died before receiving CABG, and then those who make it after surgery could be more likely to survive.

Many patients undergoing CABG do not have previous CVEs such as MI. We hypothesized that the prognostic impact of FHpCVD may differ based on the presence of prior CVEs at the time of the index operation. We found a clear distinction in the association of FHpCVD with outcomes between the two subgroups with and without prior MI or revascularization. This finding can inform future research, as it seems that among surgical patients, FHpCVD has different associations with first-time and subsequent CVEs.

### Limitations

Even though this study examined outcomes with a median follow-up of more than six years, it can be improved by a longer follow-up. FHpCVD in first-degree relatives was determined according to patient self-report. This could potentially lead to recall bias; however, studies have reported reasonable accuracy with FHpCVD self-report [27, 28], and have shown that self-reported FHpCVD is independently associated with outcomes [3, 21]. Moreover, FHpCVD is usually determined by the same method in clinical practice. Finally, as previously discussed, we were not able to statistically control for all confounding variables associated with FHpCVD and outcomes in patients undergoing CABG.

## Conclusion

This study provides an in-depth evaluation of the prognostic role of FHpCVD among patients undergoing CABG. Patients with FHpCVD were older, had more risk factors, and lower ejection fractions, but after statistical adjustments for these variables, FHpCVD had a weak association with lower all-cause mortality and no association with non-fatal CVEs; however, among patients without previous MI or PCI, those with FHpCVD experienced a significantly lower all-cause mortality, but higher non-fatal CVEs. Such discordance calls for caution in assuming a protective role for FHpCVD in patients after CABG. Future investigations are needed to further describe the prognostic significance of FHpCVD in this population.

## Data Availability

The datasets analysed in this study are available from the corresponding author upon reasonable request.

## Abbreviations

ACS: Acute coronary syndrome
CABG: Coronary artery bypass grafting
CI: Confidence interval
CVD: Cardiovascular disease
CVEs: Cardiovascular events
FHpCVD: Family history of premature cardiovascular disease
HR: Hazard ratio
IPW: Inverse probability weights
MI: Myocardial infarction
PCI: Percutaneous coronary intervention
SD: Standard deviation
SHR: Sub-distribution hazard ratio
STEMI: ST-segment elevation myocardial infarction
TIA: Transient ischemic attack

## References

[1] Lloyd-Jones DM, Nam BH, D’Agostino RB, Levy D, Murabito JM, Wang TJ (2004). Parental cardiovascular disease as a risk factor for cardiovascular disease in middle-aged adults: a prospective study of parents and offspring. JAMA.

[2] Myers RH, Kiely DK, Cupples LA, Kannel WB (1990). Parental history is an independent risk factor for coronary artery disease: the Framingham Study. Am Heart J.

[3] Chow CK, Islam S, Bautista L, Rumboldt Z, Yusufali A, Xie C (2011). Parental history and myocardial infarction risk across the world: the INTERHEART study. J Am Coll Cardiol.

[4] Arnett DK, Blumenthal RS, Albert MA, Buroker AB, Goldberger ZD, Hahn EJ (2019). 2019 ACC/AHA guideline on the primary prevention of cardiovascular disease: executive summary: a report of the American College of Cardiology/American Heart Association Task Force on Clinical Practice Guidelines. J Am Coll Cardiol.

[5] Ruttmann E, Abfalterer H, Dietl M, Wagner J, Kilo J, Grimm M (2020). Positive family history of cardiovascular disease and long-term outcomes after coronary artery bypass grafting: a genetic paradox?. Eur J Cardiothoracic Surg.

[6] Wu C, Camacho FT, Wechsler AS, Lahey S, Culliford AT, Jordan D (2012). Risk score for predicting long-term mortality after coronary artery bypass graft surgery. Circulation.

[7] Abdi-Ali A, Shaheen AA, Southern D, Zhang M, Knudtson M, White J (2016). Relation between family history of premature coronary artery disease and the risk of death in patients with coronary artery disease. Am J Cardiol.

[8] Preisler Y, Ziv-Baran T, Chorin E, Margolis G, Khoury S, Shacham Y (2018). Family history of coronary artery disease and adverse clinical outcomes in patients suffering from acute ST-segment elevation myocardial infarction. Coron Artery Dis.

[9] Karimi AA, Ahmadi SH, Davoodi S, Marzban M, Movahedi N, Abbasi K (2008). First database report on cardiothoracic surgery in Tehran Heart Center. Iran J Publ Heal.

[10] Poorhosseini H, Abbasi SH (2018). The Tehran Heart Center. Eur Heart J.

[11] Murabito JM, Pencina MJ, Nam BH, D’Agostino RB, Wang TJ, Lloyd-Jones D (2005). Sibling cardiovascular disease as a risk factor for cardiovascular disease in middle-aged adults. JAMA.

[12] Kannel WB, Feinleib M, Mcnamara PM, Garrison RJ, Castelli WP (1979). An investigation of coronary heart disease in families: the framingham offspring study. Am J Epidemiol.

[13] Shuster JJ (1991). Median follow-up in clinical trials. J Clin Oncol.

[14] Masoudkabir F, Yavari N, Pashang M, Sadeghian S, Jalali A, Poorhosseini H (2020). Effect of persistent opium consumption after surgery on the long-term outcomes of surgical revascularisation. Eur J Prev Cardiol.

[15] Van Der Weele TJ, Ding P (2017). Sensitivity analysis in observational research: Introducing the E-Value. Ann Intern Med.

[16] Mathur MB, Ding P, Riddell CA, VanderWeele TJ (2018). Web site and R package for computing E-values. Epidemiology.

[17] Canto JG, Kiefe CI, Rogers WJ, Peterson ED, Frederick PD, French WJ (2012). Atherosclerotic risk factors and their association with hospital mortality among patients with first myocardial infarction (from the national registry of myocardial infarction). Am J Cardiol.

[18] Levi A, Chezar-Azerrad C, Hasdai D, Beigel R, Gottlieb S, Eisen A (2018). Impact of self-reported family history of premature cardiovascular disease on the outcomes of patients hospitalized for acute coronary syndrome (from the Acute Coronary Syndrome Israel Survey [ACSIS] 2000 to 2013). Am J Cardiol.

[19] Ertelt K, Généreux P, Mintz GS, Brener SJ, Kirtane AJ, McAndrew TC (2014). Clinical profile and impact of family history of premature coronary artery disease on clinical outcomes of patients undergoing primary percutaneous coronary intervention for ST-elevation myocardial infarction: Analysis from the HORIZONS-AMI Trial. Cardiovasc Revascularization Med.

[20] Agarwal MA, Garg L, Lavie CJ, Reed GL, Khouzam RN (2018). Impact of family history of coronary artery disease on in-hospital clinical outcomes in ST-segment myocardial infarction. Ann Transl Med.

[21] Murabito JM, Nam BH, D’Agostino RB, Lloyd-Jones DM, O’Donnell CJ, Wilson PWF (2004). Accuracy of offspring reports of parental cardiovascular disease history: the Framingham Offspring Study. Ann Intern Med.

[22] Zeitouni M, Clare RM, Chiswell K, Abdulrahim J, Shah N, Pagidipati NP (2020). Risk factor burden and long-term prognosis of patients with premature coronary artery disease. J Am Heart Assoc.

[23] Fritz J, Shiffman D, Melander O, Tada H, Ulmer H (2017). Metabolic mediators of the effects of family history and genetic risk score on coronary heart disease-findings from the Malmö Diet and Cancer Study. J Am Heart Assoc.

[24] Winnicki M, Somers VK, Dorigatti F, Longo D, Santonastaso M, Mos L (2006). Lifestyle, family history and progression of hypertension. J Hypertens.

[25] Williamson C, Jeemon P, Hastie CE, McCallum L, Muir S, Dawson J (2014). Family history of premature cardiovascular disease: blood pressure control and long-term mortality outcomes in hypertensive patients. Eur Heart J.

[26] Cole SR, Platt RW, Schisterman EF, Chu H, Westreich D, Richardson D (2010). Illustrating bias due to conditioning on a collider. Int J Epidemiol.

[27] Silberberg JS, Wlodarczyk J, Fryer J, Ray CD, Hensley MJ (1998). Correction for biases in a population-based study of family history and coronary heart disease: the Newcastle Family History Study I. Am J Epidemiol.

[28] Silberberg JS, Wlodarczyk J, Fryer J, Robertson R, Hensley MJ (1998). Risk associated with various definitions of family history of coronary heart disease the newcastle family history study II. Am J Epidemiol.

